# The contribution of spinal glial cells to chronic pain behaviour in the monosodium iodoacetate model of osteoarthritic pain

**DOI:** 10.1186/1744-8069-7-88

**Published:** 2011-11-17

**Authors:** Devi Rani Sagar, James J Burston, Gareth J Hathway, Stephen G Woodhams, Richard G Pearson, Andrew J Bennett, David A Kendall, Brigitte E Scammell, Victoria Chapman

**Affiliations:** 1 Arthritis Research U.K. Pain Centre, University of Nottingham, Nottingham, UK; 2 School of Biomedical Sciences, University of Nottingham, Nottingham, UK; 3 Division of Orthopaedic and Accident Surgery, School of Clinical Sciences, University of Nottingham, Nottingham, UK

**Keywords:** Osteoarthritis, Microglia, Astrocytes, Central Sensitization

## Abstract

**Background:**

Clinical studies of osteoarthritis (OA) suggest central sensitization may contribute to the chronic pain experienced. This preclinical study used the monosodium iodoacetate (MIA) model of OA joint pain to investigate the potential contribution of spinal sensitization, in particular spinal glial cell activation, to pain behaviour in this model. Experimental OA was induced in the rat by the intra-articular injection of MIA and pain behaviour (change in weight bearing and distal allodynia) was assessed. Spinal cord microglia (Iba1 staining) and astrocyte (GFAP immunofluorescence) activation were measured at 7, 14 and 28 days post MIA-treatment. The effects of two known inhibitors of glial activation, nimesulide and minocycline, on pain behaviour and activation of microglia and astrocytes were assessed.

**Results:**

Seven days following intra-articular injection of MIA, microglia in the ipsilateral spinal cord were activated (p < 0.05, compared to contralateral levels and compared to saline controls). Levels of activated microglia were significantly elevated at day 14 and 21 post MIA-injection. At day 28, microglia activation was significantly correlated with distal allodynia (p < 0.05). Ipsilateral spinal GFAP immunofluorescence was significantly (p < 0.01) increased at day 28, but not at earlier timepoints, in the MIA model, compared to saline controls. Repeated oral dosing (days 14-20) with nimesulide attenuated pain behaviour and the activation of microglia in the ipsilateral spinal cord at day 21. This dosing regimen also significantly attenuated distal allodynia (p < 0.001) and numbers of activated microglia (p < 0.05) and GFAP immunofluorescence (p < 0.001) one week later in MIA-treated rats, compared to vehicle-treated rats. Repeated administration of minocycline also significantly attenuated pain behaviour and reduced the number of activated microglia and decreased GFAP immunofluorescence in ipsilateral spinal cord of MIA treated rats.

**Conclusions:**

Here we provide evidence for a contribution of spinal glial cells to pain behaviour, in particular distal allodynia, in this model of osteoarthritic pain. Our data suggest there is a potential role of glial cells in the central sensitization associated with OA, which may provide a novel analgesic target for the treatment of OA pain.

## Background

Osteoarthritis (OA) is the most prevalent joint disease and knee OA is the major cause of lower limb disability in older people worldwide [1]. The major symptoms of OA are chronic pain and disability. Current analgesic strategies for the long term treatment of OA pain have modest effects and are often associated with severe side-effects. The improved treatment of OA pain is a major unmet clinical need, which can only be addressed by a better understanding of the mechanisms that drive this chronic pain state. Although the structural changes that occur at the level of the OA joint are well characterized, the association(s) between these changes and the extent of pain experienced are ill-defined [2]. This variable link between injury and pain seen in OA patients, and the report of spreading pain and facilitation of pain responses (known as central sensitization) in OA patients [3], suggests the spinal and supraspinal [4] processing of painful inputs is altered in OA.

The aim of the present study was to investigate the cellular substrates activated in the spinal cord, a key region of pain processing and central sensitization, in an established animal model of OA pain. Intra-articular injection of the glycolysis inhibitor monosodium iodoacetate (MIA) into the rat knee produces pathology of the joint [5-8], which has similarities to that seen in human osteoarthritic joints, and also elicits pain behaviours. MIA-treated rats exhibit significant decreases in weight-bearing on the ipsilateral hind-limb [5,6] and aberrant pain responses from sites distal to the joint (secondary hyperalgesia), specifically mechanical allodynia of the ipsilateral hindpaw [6,9]. Previous work from our group has shown that the effects of MIA treatment on cartilage and subchondral bone pathology are significantly correlated with pain behaviour and innocuous mechanically-evoked responses of spinal neurones at 28 days post-injection, but not at earlier timepoints [10]. The presence of distal mechanical allodynia following joint pathology [6,9,11] and enhanced spinal neuronal activity [10] suggests mechanisms of central sensitization [12], which contribute to other chronic pain states, may be involved. Central sensitization of nociceptive processing has been investigated widely [see references in [12]]. The increased excitability observed in the dorsal horn that characterises central sensitization results from specific patterns of nociceptive input from the periphery, alterations in the dorsal horn and also increased facilitatory drive from the brainstem [13]. The establishment of central sensitization leads to tactile allodynia and the "spread" of pain hypersensitivity to healthy tissue (secondary hyperalgesia). Traditionally the establishment of central sensitization was considered a purely neuronal event. This notion was dispelled by the demonstration of a significant role for non-neuronal, glial, cells of different types in the establishment and maintenance of central sensitization particularly in neuropathic pain states [14-16]. Activation of spinal glial cells has a pivotal role in the generation and maintenance of allodynia following nerve injury [see references in [12,14-19]. In this regard, the commonality of OA pain mechanisms with neuropathic pain states is of particular note [20]. Spinal microglia [21], but not astrocytic, activation has been reported at early timepoints in the MIA model of OA pain and was associated with pain behaviour [22]. However, it is only at later time-points (day 28) that neuronal correlates of pain behaviour are observed in this model of OA pain [10]. We hypothesised that distal allodynia associated with pathological changes to the knee following MIA treatment would be associated with changes in the activation state of both microglia and astrocytes, which are also increasingly implicated in maintaining chronic pain states [23], at this later time-point.

The aim of the present study was to correlate changes in microglial activation state alongside potential changes in astrocytic reactivity both at early and late time-points in this model. The functional contribution of microglia and astrocytes to the development of chronic pain behaviour was assessed by determining the effects of the tetracycline minocycline, which attenuates microglia activation and chronic pain behaviour [19], and the cycloxygenase inhibitor nimesulide, on pain behaviour and activation of glial cells in the spinal cord in this model of OA pain.

## Results

### Spinal glial cell activation is significantly correlated with OA pain behaviour

**Figure 1:**
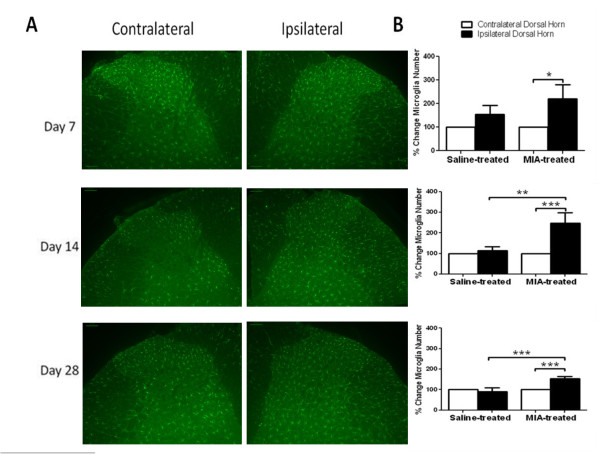
**Timecourse of spinal microglia activation in MIA-treated rats**. (A) Positively identified spinal microglia were increased in the ipsilateral spinal cord of MIA-treated rats at days 7, 14 and 28, when compared to the contralateral spinal cord. Typical dorsal sections taken from spinal cords of MIA treated rats at days 7, 14 and 28 post- injection. (B) Quantification of positively identified activated microglia at the different timepoints in both treatment groups reveals significant differences. Data are expressed as mean % of the number of activated microglial in contralateral dorsal horn (100% baseline). Statistical comparison between number of Iba-1 positive cells in ipsilateral versus contralateral spinal cord were performed using a one-way ANOVA with a Dunnett's multiple comparison test *p < 0.05, **p < 0.01, ***p < 0.001. For comparisons between treatment groups, MIA versus saline-treated rats, a Kruskall Wallis test with a Dunn's post hoc test was used. **p < 0.01, ***p < 0.001.

As previously described, intra-articular injection of MIA significantly (p < 0.001) decreased ipsilateral weight-bearing and mechanical withdrawal thresholds of the hindpaw (distal allodynia), compared to saline-treated rats, from post-injection day 7 onwards (Additional file 1, Figure S1). Numbers of Iba-1 positive, morphologically identified, activated microglia were significantly increased in the ipsilateral spinal cord at days 7, 14 and 28 in MIA-treated rats (Figure 1A), compared to the contralateral side. There was also a significant (p < 0.001) difference in the numbers of Iba-1 positive, morphologically identified as activated, microglia in the ipsilateral spinal cord of MIA-treated rats, compared to saline-treated rats, at both days 14 and 28 (Figure 1B, Additional file 2, Figure S2A). The numbers of activated microglia in the ipsilateral spinal cord was significantly correlated with distal allodynia at Day 28 (p < 0.05, 2-tailed Spearman's rank correlation, Spearman R = -0.71), but not at earlier time points (2-tailed Spearman's Rank correlation; Day 7: Spearman R = -0.11; Day 14: Spearman R = -0.35).

**Figure 2:**
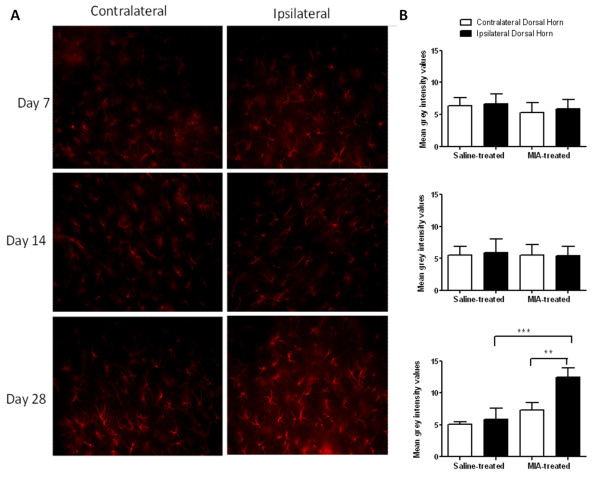
**Timecourse of spinal astrocyte activation in MIA-treated rats**. A: GFAP immunofluorescence, as a marker of astrocytes, was increased in the ipsilateral spinal cord of MIA-treated rats at day 28 post MIA treatment, compared to the contralateral spinal cord. B: Data are expressed as mean grey intensity of spinal GFAP immunofluorescence in ipsilateral and contralateral dorsal horn. Statistical comparison between GFAP immunofluorescence in ipsilateral versus contralateral spinal cord were performed using a two-way ANOVA with a Bonferroni post hoc test: **p < 0.01, ***p < 0.001.

The potential activation of spinal astrocytes was also investigated in the MIA model. Increases in GFAP immunofluorescence, indicative of astrocyte activation, were only observed at day 28 in MIA-treated rats. GFAP immunofluorescence was significantly increased in the ipsilateral dorsal horn of MIA-treated rats, compared to the contralateral spinal cord and the ipsilateral spinal cord of saline-treated rats at this timepoint (Figure 2, Additional file 2, Figure S2B). At this timepoint, GFAP immunofluorescence in the ipsilateral dorsal horn of the spinal cord was significantly correlated with distal allodynia (p < 0.01, 2-tailed Spearman's rank correlation, Spearman R = -0.84). To provide further supportive evidence for the activation of astrocytes in this model of OA pain, expression of GFAP mRNA in the spinal cord of MIA-treated was also studied. GFAP mRNA was elevated in the ipsilateral and contralateral spinal cord in MIA-treated rats, compared to saline-treated rats at day 28. Although the magnitude of elevation of GFAP mRNA was comparable in the ipsilateral and contralateral spinal cord of MIA-treated rats, compared to saline controls, significance was only reached for the contralateral spinal cord (Additional file 3, Figure S3). These data suggest there may be bilateral changes in GFAP reactivity at later time points in this model.

### Inhibition of spinal glial cell activation has sustained inhibitory effects on OA pain behaviour

**Figure 3:**
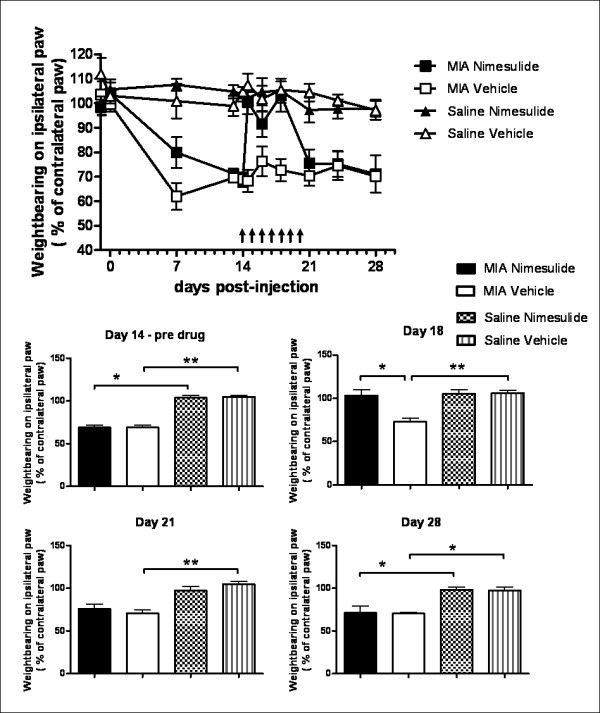
**Nimesulide attenuates established weight bearing deficits in MIA-treated rats**. A: Timecourse of the effects of repeated oral administration of nimesulide (10 mg.kg^-1^; days 14-20) on weight bearing on the ipsilateral hindpaw in MIA and saline-treated rats. Arrows indicate the timing of drug administration. B: Histograms comparing the effects of repeated oral administration of nimesulide before (day 14), during (day 18) and after (day 21 and 28) drug administration. Data are expressed as mean ± SEM. Statistical analyses were performed using a Kruskall-Wallis test with a Dunn's post-hoc test: *p < 0.05, **p < 0.01.

**Figure 4:**
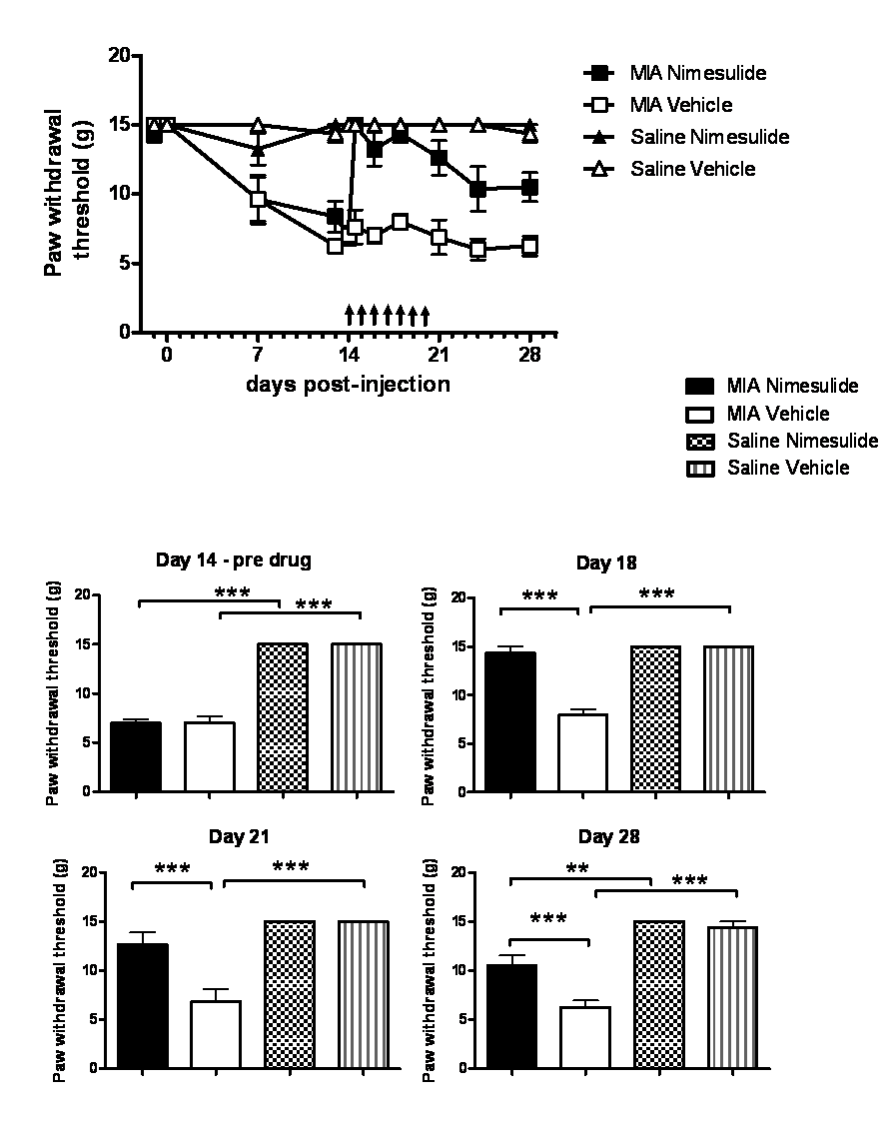
**Nimesulide attenuates established allodynia in MIA-treated rats**. A: Timecourse of the effects of repeated oral administration of nimesulide (10 mg.kg^-1^; days 14-20) or vehicle on mechanical withdrawal thresholds of the ipsilateral hindpaw in MIA and saline-treated rats. Arrows indicate the timing of drug administration. B: Histograms comparing the effects of repeated oral administration of nimesulide before (day 14), during (day 18) and after (day 21 and 28) drug administration. Data are expressed as mean ± SEM. Statistical analyses were performed using a Kruskall-Wallis test with a Dunn's post-hoc test. *p < 0.05, **p < 0.01, ***p < 0.001.

**Figure 5:**
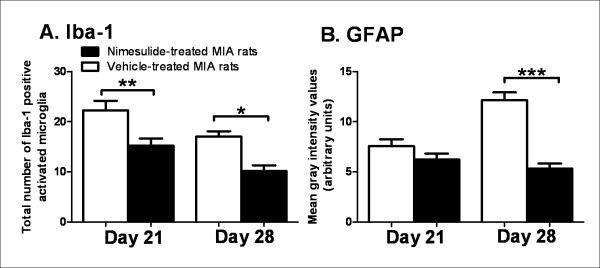
**Nimesulide attenuates microglia and astrocyte activation in MIA-treated rats**. A: Repeated administration of nimesulide (10 mg.kg^-1^; days 14-20) significantly attenuated MIA-induced increases in Iba-1 positive activated microglia in the ipsilateral dorsal horn on day 21 and day 28, compared to vehicle-treated rats. B: Repeated administration of nimesulide (10 mg.kg^-1^; days 14-20) did not alter GFAP immunofluorescence on day 21. Nimesulide treatment did, however, significantly attenuate MIA-induced increases in GFAP immunofluorescence in the ipsilateral dorsal horn at day 28, compared to vehicle-treated rats. Data are expressed as mean ± SEM. Statistical analyses comparing effects of nimesulide treatment versus vehicle were performed using a 1 way ANOVA with a Bonferroni post hoc test: *p < 0.05, **p < 0.01. ***p < 0.001.

Oral administration of nimesulide (days 14-20 post-MIA injection) significantly attenuated MIA-induced decreases in weight bearing (Figure 3) and distal allodynia (Figure 4) in MIA-treated rats, compared to MIA-treated rats receiving vehicle, throughout the period of drug administration. At day 21, just after dosing with nimesulide had ceased, the number of Iba-1 positive activated microglia in the ipsilateral dorsal horn of nimesulide-treated MIA rats was significantly lower than the number present in MIA-treated rats which received vehicle (Figure 5A). Consistent with the time-course of astrocyte activation described above, GFAP immunofluorescence was not significantly altered in the ipsilateral spinal cord of MIA-treated rats at day 21, compared to both contralateral spinal cord and saline controls. Repeated daily treatment with nimesulide did not alter GFAP immunofluorescence of the ipsilateral spinal cord of MIA-treated rats at day 21 (Figure 5B).

Once dosing with nimesulide had ceased, changes in weight bearing returned to pre-nimesulide values in MIA treated rats from day 21 onwards (Figure 3). By contrast, the inhibitory effects of nimesulide on distal allodynia persisted even once dosing had ceased and these inhibitory effects were maintained from day 21 until the end of the study (day 28) (Figure 4). Since nimesulide treatment has sustained inhibitory effects on distal allodynia in MIA-treated rats, we then investigated the potential longer-term effects of repeated daily treatment (days 14-20) with nimesulide on microglia and astrocyte activation at day 28 in MIA-treated rats. In this case, the earlier treatment with nimesulide significantly decreased the number of Iba-1 positive activated microglia (Figure 5A) and the level of GFAP immunofluorescence (Figure 5B) in the ipsilateral spinal cord at day 28 of MIA-treated rats, compared to MIA-treated rats receiving vehicle. Nimesulide treatment did not alter MIA-induced joint pathology at day 28 (data not shown).

**Figure 6:**
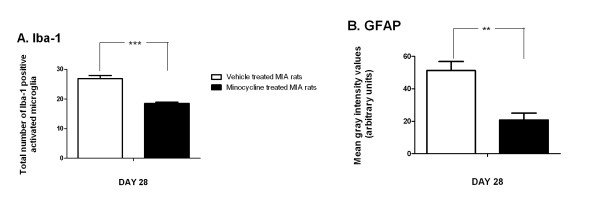
**Minocycline attenuates microglia and astrocyte activation in MIA-treated rats**. A: Minocycline treatment (30 mg.kg^-1^) significantly attenuated MIA-induced increases in Iba-1 positive activated microglia in the ipsilateral dorsal horn at day 28, compared to the effects of vehicle treatment in MIA-injected rats. B: Minocycline treatment (30 mg.kg^-1^) significantly attenuated MIA-induced increases in GFAP immunofluorescence in the ipsilateral dorsal horn at day 28, compared to MIA-treated rats which received vehicle. Data are expressed as mean ± SEM. Statistical analyses comparing effects of minocycline treatment versus vehicle were performed using a Student's t test: **p < 0.01. ***p < 0.001.

To further confirm the association between pain behaviour and spinal glial activation in this model of OA pain, we determined the effects of another inhibitor of glial cell activation, minocycline, on pain behaviour and glial cell activation. Minocycline treatment (30 mg.kg^-1^) significantly (P < 0.01) attenuated pain behaviour (mechanical withdrawal thresholds: MIA plus vehicle: 4 +/- 1.25 grams; MIA plus minocycline 13.75 +/- 1.41 grams) and the number of Iba-1 positive activated microglia in the ipsilateral dorsal horn of MIA-treated rats, compared to the effect of vehicle, at day 28 (Figure 6A). Furthermore, minocycline treatment significantly reduced the MIA-induced increase in GFAP immunofluorescence in the ipsilateral spinal cord of MIA treated rats, compared to the effect of vehicle at day 28 (Figure 6B).

## Discussion

Here we demonstrate that the MIA model of OA pain is associated with a time dependent activation of microglia and, for the first time, the later activation of astrocytes in the spinal cord. The timecourse of activation of astrocytes was slower, compared to microglia, and significant changes in ipsilateral GFAP immunofluorescence were only observed at day 28 post-MIA injection. As previously described [10] intra-articular injection of MIA was associated with significant changes in weight bearing and decreases in hindpaw mechanical withdrawal thresholds, indicative of distal allodynia. Significant levels of distal allodynia and increases in the number of activated microglia in the ipsilateral spinal cord of MIA-treated rats were observed at day 7 post-MIA injection. In keeping with two recent studies [21,22], the numbers of activated microglia present in the ipsilateral spinal cord were further increased at day 14, and in our hands by day 28 the numbers of activated microglia were declining.

The present study used intra-articular injection of MIA as a model of joint pathology and pain associated with OA. The dose of MIA employed has previously been shown to produce joint pathology which has features consistent with that seen in patients with endstage OA. Importantly, this model of OA pain is associated with changes in weight bearing and distal (hindpaw) allodynia and, therefore, has translational relevance to both pain on load-bearing and referred pain, both of which are experienced by OA sufferers. Although pain behaviour is apparent at early timepoints in the MIA model, we have shown that it is only at later timepoints (day 28 post MIA-injection) that evoked responses of spinal neurones are facilitated and significantly correlated with pain behaviour [10], implicating a contribution of central sensitization to aberrant pain responses at these later stages. The presence of distal allodynia in the MIA model may reflect the presence of nerve damage, as previously described [20**]**, changes in the receptor expression in peripheral nerves innervating the joint [24], and a potential role of infiltrating immune cells and satellite glia [17], all of which may contribute to the increase spinal excitability seen in this model. Spinal COX-2 expression plays a crucial role in central sensitization in models of pain [25,26]; the increase in spinal COX-1 and COX-2 mRNA in MIA-treated rats [27] supports a contribution of central sensitization to OA pain. Our current work further advances this hypothesis through the demonstration of a time-dependent activation of microglia and astrocytes in the spinal cord in this model of OA pain.

The activation of microglia and astrocytes in the spinal cord has been widely reported in different models of chronic pain, and their contribution to pro-inflammatory spinal mechanisms of central sensitization has been described [see references in [12]]. The presence of activated microglia and astrocytes in the spinal cord in this model of OA pain suggests these cells may also contribute to the associated pain behaviour. In models of neuropathic pain, microglia are activated in the spinal cord at earlier timepoints compared to astrocytes [see references in [28]]. This temporal profile, plus the evidence from pharmacological studies using minocycline, support the notion that microglia have a prominent role in the development, but not the maintenance, of hyperalgesia and allodynia in models of neuropathic pain [19]. Thus, the early activation of microglia followed by the later activation of astrocytes that we describe in the MIA model of OA pain is consistent with that reported in neuropathic pain states [16,19,29] and the report that microglia activation is associated with all phases of inflammation whereas astrocyte activation is only observed during subacute and chronic phases of inflammation [30]. These data suggest there is a neuronal-glial cascade of activation which is common to different models of chronic pain. In this regard it is noteworthy that the presence of activated microglia and astrocytes in the MIA-model was observed at later timepoints than that reported for models of neuropathic and inflammatory pain, this slower onset may reflect the development of structural changes within the joint.

Microglia are activated in response to numerous cues, many of which can be released from primary sensory afferents [17,31]. Although distal allodynia and activation of spinal microglia was evident from day 7 post-MIA injection, it was only at day 28 that levels of activated microglia were significantly correlated with distal allodynia. Consistent with the profile of glial cell activation in other models of chronic pain, GFAP immunofluorescence was only significantly increased at day 28, at which timepoint GFAP immunofluorescence was also significantly correlated with distal allodynia. The activation of spinal astrocytes coincides with the facilitation of evoked responses of spinal neurones and the significant correlation of distal allodynia with cartilage and bone pathology previously reported [10].

To assess the potential contribution of the activated microglia and astrocytes to the MIA-induced pain behaviour, we evaluated the effects of two different classes of drug, the COX inhibitor nimesulide [32,33] and the tetracycline minocycline [19], on pain responses. Treatment with both of these drugs attenuated both pain behaviour and the activation of microglia and astrocytes in the spinal cord in this model of OA pain. Previously, nimesulide has been shown to attenuate microglia activation in models of neuroinflammation [32,33], which is likely to be an indirect effect as a result of COX inhibition [34]. Minocycline has well-described effects on microglia activation [see refs in 19], however there are also reports that minocycline can have direct effects on dorsal root ganglia neurones [35] and the trafficking of immune cells into the dorsal root ganglia [36], both of which may contribute to the effects reported herein.

Our data support the recent report that pain behaviour is associated with the activation of spinal microglia at earlier timepoints in this model of OA pain [22] and advance our understanding of the contribution of glial cells, to include a role of astrocytes, at later stages in this model of OA pain. Repeated dosing with nimesulide (day 14-20) significantly decreased pain behaviour and the number of activated microglia in the ipsilateral spinal cord of MIA-treated rats as assessed at day 21 post MIA-injection. At this timepoint, GFAP immunofluorescence in the spinal cord was not altered in MIA-treated rats per se, and nimesulide treatment had no effect on this marker of astrocyte activation. Given the robust effects of nimesulide on pain behaviour and microglia activation, it was of interest to determine whether the inhibitory effects of nimesulide had any sustained effects on pain behaviour and glial cell activation. Following cessation of drug treatment, changes in weight bearing on the MIA-injected joint returned to pre-nimesulide values, by contrast there was a sustained inhibition of distal allodynia and levels of activated microglia and astrocytes at day 28. Given that nimesulide is cleared within 40 h post-drug administration in the rat [34], the sustained effects of nimesulide are likely to arise from the modulation of pain mechanisms rather than residual drug action. The dissociation between the effects of nimesulide on the two pain behaviours once drug treatment has ceased, suggests that changes in weight bearing and distal allodynia are mediated by different mechanisms at this later timepoint in MIA-treated rats. Alterations in spinal glial glutamate transport are implicated as one of the underlying mechanisms of allodynia in other models of chronic pain, and inhibition of astrocyte activation with propentofylline modulates both pain behaviour and glial glutamate promoter activation [37]. Whether similar mechanisms contribute to the chronic pain responses seen in the MIA model, and the sustained effects of nimesulide on mechanical allodynia, compared to weight bearing described herein, remain to be determined.

Our pharmacological data support an important contribution of activated spinal microglia and astrocytes to distal allodynia at later timepoints in this model. Indeed, the lack of effect of nimesulide treatment on joint pathology suggests peripheral effects are unlikely to overtly contribute to the inhibitory effects of nimesulide on pain behaviour. It is of particular note that although astrocytes are quiescent during the period over which nimesulide treatment was administered (14-20 days) in the MIA-treated rats, this treatment did attenuate the later activation of astrocytes at day 28. The role of microglia signalling to astrocytes and their subsequent activation is well established [38-40], thus it is feasible that the effects of nimesulide on astrocyte activation arise as a result of the inhibition of microglia activation during the earlier stages of the model.

In conclusion, the present study has demonstrated the presence of activated microglia and astrocytes in the spinal cord in the MIA model of OA pain. The significant correlation between distal allodynia and activation of spinal glial cells, plus the attenuation of pain behaviour by inhibitors of glial cell activation, provides the first evidence that both spinal microglia and astrocytes contribute to behavioural pain responses in this model of OA pain. The association of astrocytes with aberrant pain behaviour was only evident at later time-points, which corresponds to the manifestation of spinal neuronal facilitation in this model [10]. Our preclinical data support recent clinical evidence that OA pain is accompanied with central sensitization [4] and implicate a contribution of activated glial cells to these chronic pain mechanisms.

## Methods

Rats were purchased from Charles River U.K. All studies were carried out in accordance with UK Home Office Animals (Scientific Procedures) Act (1986) and follow the guidelines of the International Association for the Study of Pain. A total of 91 male Sprague Dawley rats weighing 160-190 g were used for these studies (n = 32 rats for behavioural pharmacology studies, n = 59 rats for immunohistochemical studies).

### Intra-articular injections and behavioural testing

Adult male Sprague Dawley rats (160-190 g) were anesthetised with isoflurane (1.5-2% in 50% N_2_O-50% O_2_) and received a single intra-articular injection of monosodium iodoacetate (MIA; 1 mg/50 μl; Sigma U.K.) in saline through the infra-patellar ligament of the left knee. The dose of MIA was based on the previous literature [5,41,42]. Control animals received a single intra-articular injection of saline (50 μl).

Baseline behavioural measurements were taken prior to intra-articular injection (postoperative day (PO) 0) and then from PO day 7 onwards. Effects of MIA or saline injection on weight-distribution through the left (ipsilateral) and right (contralateral) knee were assessed using an incapacitance tester (Linton Instrumentation, U.K.) between post-operative days 7-28. Hind-paws were placed on separate sensors and the force (in grams) exerted by each hind limb was calculated and averaged over a period of 3 seconds as previously described [43,44]. Each data point was taken as the mean of three 3 sec readings. The development of mechanical allodynia was assessed using von Frey monofilaments (Semmes-Weinstein monofilaments of bending forces 1, 1.4, 2, 4, 6, 8, 10 and 15 g) as previously described [10]. Von Frey monofilaments were applied to the plantar surface of both hind-paws for a 3 sec period. Once a withdrawal reflex was established, the paw was retested with the next descending von Frey monofilament until no response occurred. The lowest weight of monofilament which elicited a withdrawal reflex was noted as the paw withdrawal threshold (PWT).

### Drug treatment

The effects of repeated (day 14-21 post MIA-injection) oral administration of nimesulide (10 mg.kg-^1^; Tocris, U.K.; n = 8 rats per group), or vehicle (2% methylcellulose in distilled water; n = 8 rats per group), on changes in weight bearing and mechanical allodynia were assessed for 7 days in MIA and saline-treated rats. Weight bearing and mechanical allodynia were assessed before nimesulide administration and at 40 min intervals post-drug administration. To determine whether there were any longer-term effects of repeated dosing with nimesulide on pain behaviour, which outlast the dosing schedule, behavioural testing was continued from days 21-28. At day 28 rats were killed and spinal cord and joints were collected for gene expression studies and joint histology, respectively. In a separate group of rats, the effects of the nimesulide treatment on the numbers of activated microglia and astrocytic activation in the spinal cord of MIA- and saline-treated rats were determined (n = 5 rats per group). Rats were perfused at days 21 or day 28 post-MIA injection and spinal cords were removed and prepared for immunohistochemistry (see below). In a final series of experiments, the effects of another glial cell inhibitor minocycline (30 mg.kg-^1 ^daily treatment from day 14-28) on pain behaviour (as described above) was determined in MIA-treated rats. At day 28 post MIA-injection, rats were perfused and spinal cords were collected for immunohistochemical analysis.

### Immunohistochemistry

Rats were overdosed with sodium pentobarbital and transcardially perfused with saline (0.9%) followed by 4% paraformaldehyde (PFA; Sigma, U.K). The lumbar spinal cord was removed, post-fixed (4% PFA for 4 h) and stored in 30% sucrose in 0.1 M phosphate buffer/0.02% sodium azide solution at 4 C. Immunohistochemical staining was performed on 40 μm free-floating cryosections of L3/L4/L5 spinal cord (n = 4-6 rats per group, 5-6 individual spinal sections per animal). Microglial cells were stained using Iba-1 [45] and astrocytes were stained using GFAP [46]. Sections were blocked for 1 hr in 0.1 M phosphate buffer saline containing 3% normal goat serum and 0.3% Triton X-100 at room temperature. Sections were then incubated at 4°C for 72 h with rabbit α-Iba-1 (Wako, Japan) diluted 1:1000 in Trizma Triton X-100 buffered saline (TTBS) or incubated at room temperature 20-22°C for 18 h with mouse anti-GFAP (Thermo-Fisher, Leicestershire, UK) diluted 1:100 in TTBS Five 10 min washes in 0.1 M phosphate buffer (PB) were carried out between all subsequent steps. Sections were incubated for 2 h at room temperature with Alexafluor 488 conjugated goat α-rabbit secondary antibody (Molecular probes, Oregon) diluted 1:300 in TTBS (for Iba-1) or with Alexafluor 568 conjugated goat α-mouse secondary antibody (Molecular probes, Oregon) diluted 1:300 in TTBS (for GFAP). Sections were then mounted on gelatinized microscope slides, air-dried overnight at room temperature in the dark and coverslipped using Fluromount (Sigma).

### Quantification of glial activation

Iba-1 immunostaining was visualised using a 20 × 0.4 NA objective lens on a Leica DMIRE2 fluorescence microscope, running Volocity 5.5 (PerkinElmer) equipped with a Hamamatsu Orca C4642-95 camera. Images were acquired using a typical exposure time of 750 ms. Total numbers of positively identified activated microglia expressing Iba-1 were counted manually in both ipsi- and contralateral quadrants of individual sections (Additional file 4, Figure S4). Microglia were defined as activated if they displayed a clearly swollen cell body with reduced processes, these differ from normal or resting microglia where cell bodies are largely absent and large ramified processes are displayed. Assessment was performed by two independent blinded investigators, who quantified total number of activated microglia. For further analysis (Figure 1), numbers of activated microglia on the ipsilateral side were expressed as a percentage of the number on the contralateral side.

Images for astrocyte (GFAP) grey intensity calculation were produced via capture on a Leica DMRB fluorescence microscope using a 40X 1.25NA oil immersion objective lens Images were acquired using Openlab (PerkinElmer) to control a Hamamamtsu Orca C4642-95 camera. For quantification of GFAP, single-plane images of the superficial dorsal horn of the spinal cord were acquired on the pre-described system using an identical exposure time of 100 ms. Background fluorescence was measured by taking an image of an area within the sample, using the parameters described above, which contained no labelled structures that was then subtracted from all images using IMAGE J (NIH open software with Macbiophotonics plugins). Following background subtraction, mean fluorescence grey intensity was determined for each image using IMAGE J. All image analysis, cell counts and fluorescence measurements were performed "off-line" on captured images taken from stained sections. All and counts were independently verified by a second experimentor.

### RNA extraction and cDNA synthesis

Ipsilateral and contralateral spinal cord samples were dissected from MIA and saline-treated rats (n = 8 rats per group) and were frozen. Samples were homogenized in 2 ml of ice cold Tri reagent (Sigma-Aldrich, UK) and RNA purified according to the manufacturers' instructions. For cDNA synthesis, 100 ng of total RNA was reverse transcribed using M-MLV reverse transcriptase (Invitrogen) in a total reaction volume of 20 μl, as per the manufacturers' instructions. Reactions were incubated for 10 min at 25°C, 50 min at 37°C and the reaction terminated by incubation at 70°C for 15 min.

### Taqman quantitative real time polymerase chain reaction

Gene expression was quantified utilising the relative standard curve method, based on Taqman quantitative real time polymerase chain reaction (qRT-PCR), as previously described [47]. Primers and probes were obtained from published work (GFAP - [48], β-actin - [49]), and synthesised by MWG Biotech, (Germany). Data are expressed as a ratio of gene expression levels with reference to β-actin. GFAP forward primer - 5- TGGCCACCAGTAACATGCAA-3, reverse primer - 5-CAGTTGGCGGCGATAGTCAT-3, Taqman probe - 5-CAGACGTTGCTTCCCGCAACGC-3. Β-actin forward primer - 5- AGGCCATGTACGTAGCCATCCA-3, reverse primer - 5- TCTCCGGAGTCCATCACAATG-3, Taqman probe - 5- TGTCCCTGTATGCCTCTGGTCGTACCAC -3.

### Histology

Joint histology was conducted as previously described [10]. MIA- and saline-treated joints (day 28) were fixed in 10% formal saline and decalcified in an aqueous ethylenediaminetetraacetic acid (EDTA) solution (14% in distilled water; pH 7.0, 20°C). Samples were paraffin embedded and 5-8 μM sections of the central portion of the knee joint, in the coronal plane, were stained by safranin-O fast green to show matrix proteoglycan and overall joint morphology. Medial and lateral knee compartment tibial plateaux cartilage, tibial subchondral bone and joint synovium were scored as previously described [10].

### Statistical Analyses

Statistical analyses comparing effects of MIA-treatment versus saline-treatment on weight-bearing and paw withdrawal thresholds were carried out using a 2-way ANOVA with a Bonferroni post hoc test. Statistical analyses comparing effects between treatment groups (nimesulide-treated versus vehicle-treated on weight bearing and paw withdrawal thresholds) were performed using a Kruskall-Wallis test with a Dunn's post hoc test and area under the curve analysis with a Mann Whitney test. Statistical analyses comparing the effect of minocycline versus vehicle on pain behaviour at day 28 post MIA-injection was performed using a Student's unpaired t Test. Statistical analyses of GFAP intensity used either a one-way or two-way ANOVA (as listed in figure legends) with a Bonferroni post hoc test. Statistical analyses of changes in number of activated microglia in the spinal dorsal horn of MIA versus saline-treated rats were performed using a Kruskall Wallis test with a Dunn's post hoc test. Statistical analysis of gene expression studies was performed using a Student's unpaired t Test. Correlations were performed using the 2-tailed Spearman's rank correlation.

## Competing interests

The authors declare that they have no competing interests.

## Authors' contributions

DRS: conducted behavioural and pharmacological studies and helped draft the manuscript; JB conducted immunohistochemistry and helped draft the manuscript; GJH participated in the study design and coordination and helped draft the manuscript; SGW conducted the molecular biology; RGP conducted the histology; AJB participated in the study design and coordination; DAK participated in the acquisition of funds, study design and coordination and drafting of the manuscript; BES participated in the study design and coordination, and drafting of the manuscript; VC participated in the acquisition of funds, study design and coordination, and drafting of the manuscript. All authors read and approved the final manuscript.

## Supplementary Material

Additional file 1 **Figure S1: MIA-induced pain behaviour**. Intra-articular injection of MIA (1 mg/50 μl) produced significant decreases in (A) weight bearing on ipsilateral hind paw and (B) hindpaw mechanical withdrawal thresholds in the ipsilateral limb of rats compared to saline-treated rats. Data are expressed as mean ± SEM. Statistical analyses comparing MIA and saline-treated rats were performed using a two way ANOVA with a Bonferroni post hoc test, ***p < 0.001. Click here for file

Additional file 2 **Figure S2: Positively identified spinal microglia and GFAP immunofluorescence in the saline-treated rats**. A: Positively identified spinal microglia in the ipsilateral and contralateral spinal cord of saline-treated rats at day 28. B: GFAP immunofluorescence in the ipsilateral and contralateral spinal cord of saline-treated rats at day 28. Click here for file

Additional file 3 **Figure S3: Spinal GFAP gene expression in MIA-treated rats**. GFAP mRNA in the ipsilateral and contralateral spinal cord of saline and MIA-treated rats at day 28. Data are normalised to levels of β-actin and expressed as mean ± SEM. Statistical comparison between MIA and saline-treated rats was performed using a Student's unpaired t test. Click here for file

Additional file 4 **Figure S4: Schematic of the areas of spinal cord used for quantification**. Lumbar sections 3-5 of the spinal cord, red box (illustrates the area of analysis for Iba-1) and the blue box (illustrates the area of analysis for GFAP). Note images were captured from both sides of the spinal cord for microglia and astrocytes. Adapted from: Molander, C. and Grant, G., 1995, spinal cord cytoarchitecture. In G. Paxinoa (Ed), The Nervous System, Second Edition, Academic Press, San Diego. Click here for file
