# Supplementary figures and images for: The contribution of spinal glial cells to chronic pain behaviour in the monosodium iodoacetate model of osteoarthritic pain
Source: Mol Pain. 2011 Nov 17;7:88. doi: 10.1186/1744-8069-7-88 (PMC3271989; doi:10.1186/1744-8069-7-88)

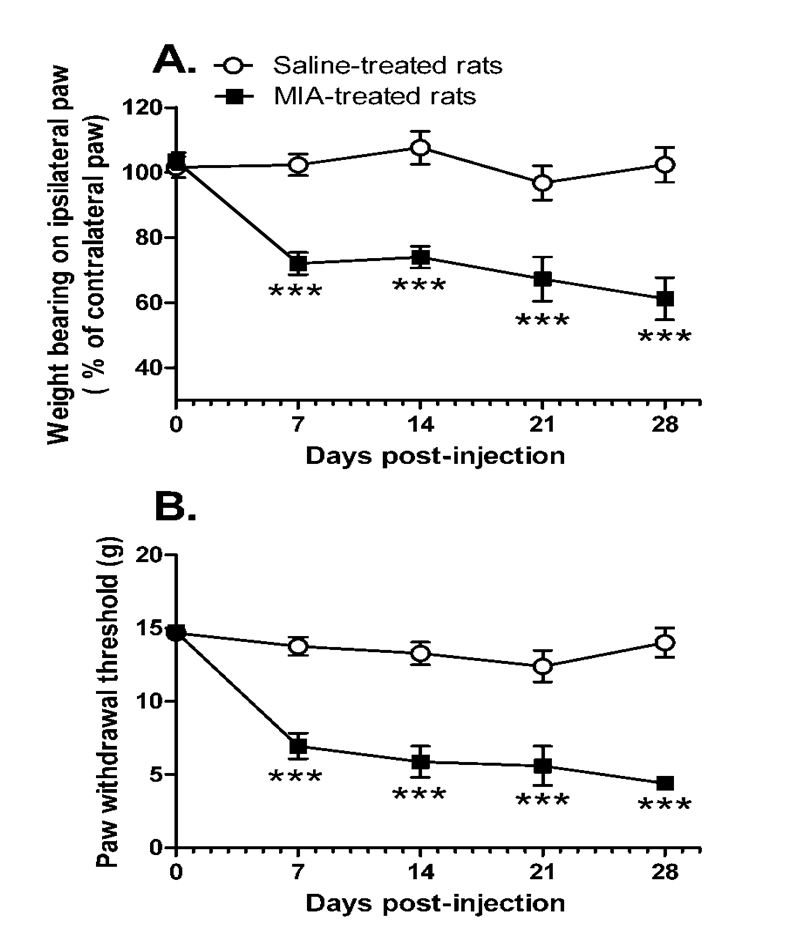

Supplement: Additional file 1 — Figure S1: MIA-induced pain behaviour. Intra-articular injection of MIA (1 mg/50 μl) produced significant decreases in (A) weight bearing on ipsilateral hind paw and (B) hindpaw mechanical withdrawal thresholds in the ipsilateral limb of rats compared to saline-treated rats. Data are expressed as mean ± SEM. Statistical analyses comparing MIA and saline-treated rats were performed using a two way ANOVA with a Bonferroni post hoc test, ***p < 0.001. [file 1744-8069-7-88-S1.TIFF]

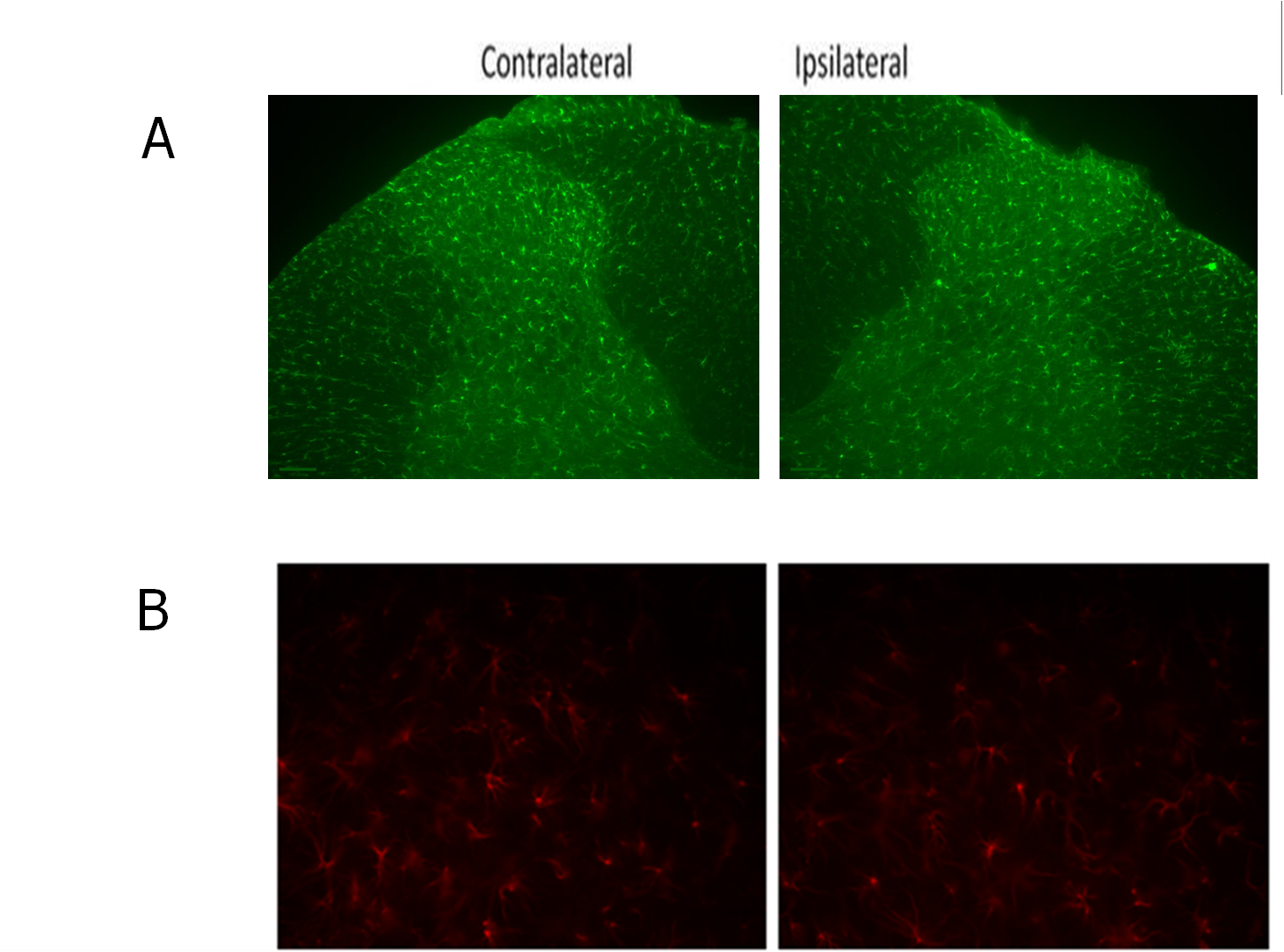

Supplement: Additional file 2 — Figure S2: Positively identified spinal microglia and GFAP immunofluorescence in the saline-treated rats. A: Positively identified spinal microglia in the ipsilateral and contralateral spinal cord of saline-treated rats at day 28. B: GFAP immunofluorescence in the ipsilateral and contralateral spinal cord of saline-treated rats at day 28. [file 1744-8069-7-88-S2.TIFF]

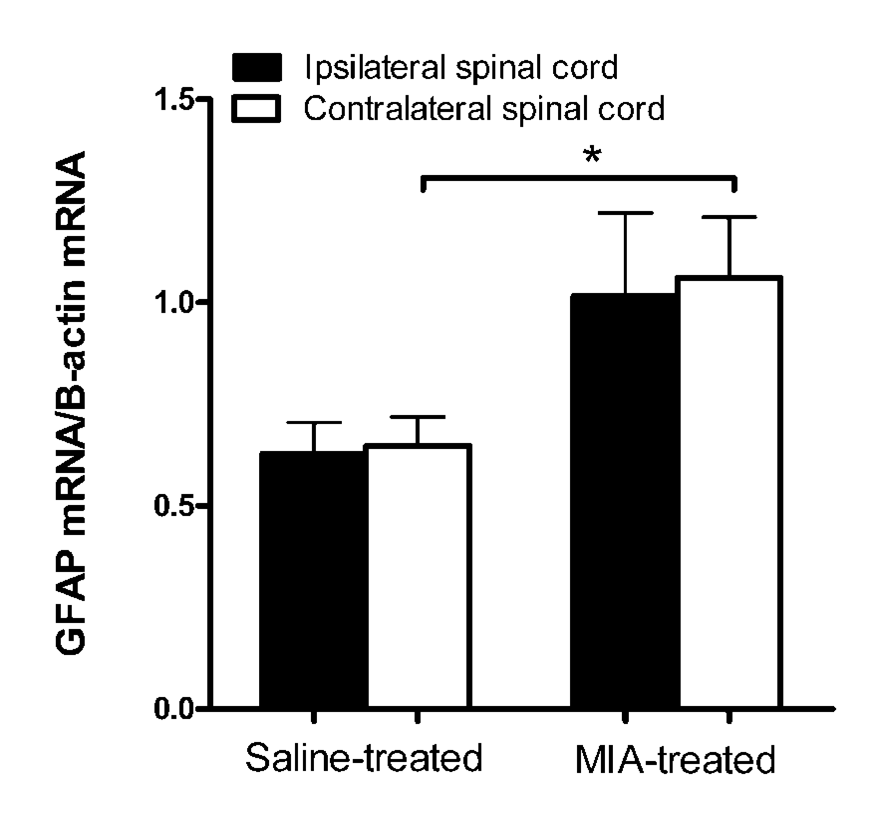

Supplement: Additional file 3 — Figure S3: Spinal GFAP gene expression in MIA-treated rats. GFAP mRNA in the ipsilateral and contralateral spinal cord of saline and MIA-treated rats at day 28. Data are normalised to levels of β-actin and expressed as mean ± SEM. Statistical comparison between MIA and saline-treated rats was performed using a Student's unpaired t test. [file 1744-8069-7-88-S3.TIFF]

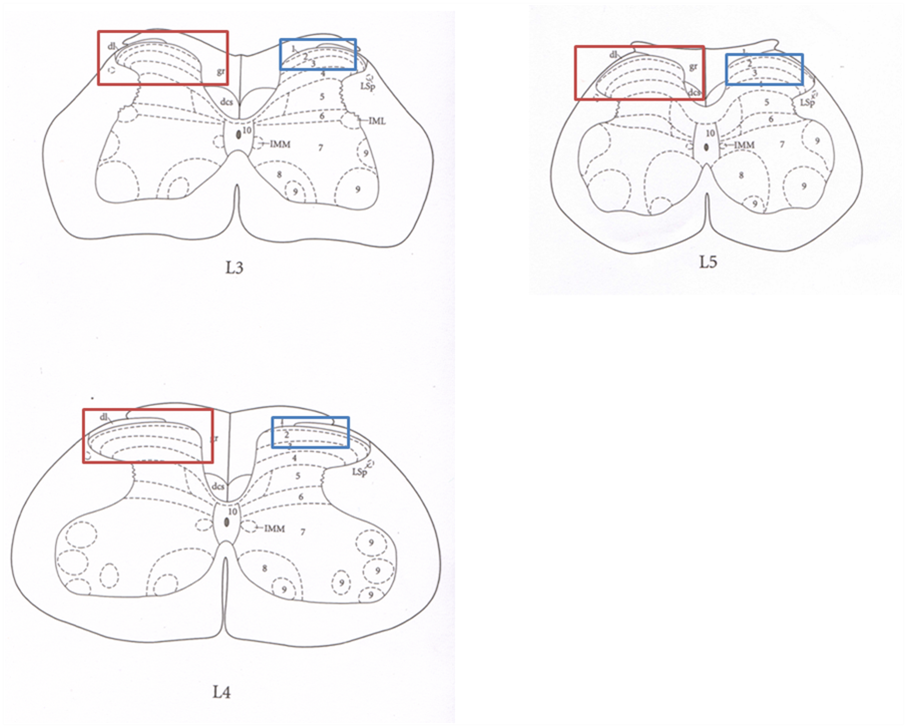

Supplement: Additional file 4 — Figure S4: Schematic of the areas of spinal cord used for quantification. Lumbar sections 3-5 of the spinal cord, red box (illustrates the area of analysis for Iba-1) and the blue box (illustrates the area of analysis for GFAP). Note images were captured from both sides of the spinal cord for microglia and astrocytes. Adapted from: Molander, C. and Grant, G., 1995, spinal cord cytoarchitecture. In G. Paxinoa (Ed), The Nervous System, Second Edition, Academic Press, San Diego. [file 1744-8069-7-88-S4.TIFF]
